# Collecting data in the home laboratory: evolution of X-ray sources, detectors and working practices

**DOI:** 10.1107/S0907444913013619

**Published:** 2013-06-18

**Authors:** Tadeusz Skarzynski

**Affiliations:** a XRD, Agilent Technologies, 10 Mead Road, Yarnton, Oxfordshire, England

**Keywords:** macromolecular crystallography, X-ray hardware, data collection

## Abstract

While the majority of macromolecular X-ray data are currently collected using highly efficient beamlines at an ever-increasing number of synchrotrons, there is still a need for high-performance reliable systems for in-house experiments. In addition to crystal screening and optimization of data-collection parameters before a synchrotron trip, the home system allows the collection of data as soon as the crystals are produced to obtain the solution of novel structures, especially by the molecular-replacement method, and is invaluable in achieving the quick turnover that is often required for ligand-binding studies in the pharmaceutical industry. There has been a continuous evolution of X-ray sources, detectors and software developed for in-house use in recent years and a diverse range of tools for structural biology laboratories are available. An overview of the main directions of these developments and examples of specific solutions available to the macromolecular crystallography community are presented in this paper, showing that data collection ‘at home’ is still an attractive proposition complementing the use of synchrotron beamlines.

## Abstract 2

Recent developments in X-ray crystallographic hardware related to structural biology research are presented and discussed.

## 1. Introduction

Out of over 75 000 crystal structures of macromolecules deposited in the Protein Data Bank (PDB) by October 2012, about 72% were solved with the use of synchrotron radiation. The proportion of structure depositions based on synchrotron data has steadily increased since the first experiments were carried out at Stanford (Phillips *et al.*, 1976 ▶) and accounts for about 85% of all depositions in the last 3–4 years. However, often only the data sets used in the final stages of model building and refinement are quoted in publications and the PDB, which may bias the statistics. A survey carried out by Agilent Technologies at the IUCr Congress in Madrid in 2011, based on replies from members of 48 different macromolecular crystallography (MX) laboratories, indicated that approximately 35% of all data sets are collected in-house. Many of these initial data sets are simply replaced by high-resolution synchrotron data sets later in the structure-determination process.

Some research groups perform all of their tests and data collections on synchrotron beamlines, especially if the synchrotron is close by and access to the beam is easy. In many laboratories the home source is only used for the initial evaluation of diffraction properties of crystals, screening for heavy-atom derivatives, testing crystals before synchrotron trips and ligand-binding studies. On the other hand, there are MX teams that can only occasionally visit a synchrotron facility or send samples for data collection. In these laboratories, the majority of the research is still carried out on in-house systems. Out of about 7600 structures deposited in the PDB in 2011, over 1100 were produced using home-laboratory systems.

## 2. Laboratory X-ray sources

All X-ray experiments in the early years of macromolecular crystallography were carried out using sealed-tube instruments with X-ray tube based on the original Coolidge design from 1913 (Coolidge, 1930 ▶). Although the X-ray beam generated by these sources was sufficient to obtain good diffraction data from crystals of small chemical molecules, it was often too weak to produce acceptable data from protein crystals. Increasing the power load on the stationary anode above a certain threshold (about 1 W per 1 µm of electron-beam diameter) results in melting of the anode material (Arndt, 1990 ▶). Therefore, to help dissipate heat, the concept of a rotating anode was introduced, firstly to generate more powerful X-rays for medical diagnostic purposes and then, from the middle of the 20th century, to produce a much stronger X-ray beam for crystallographic research. For many years, rotating-anode generators such as the Rigaku RU-H2R and Nonius FR591 were used as workhorses in almost all structural biology laboratories. The next significant step in the pursuit of even brighter home-laboratory sources was made by the introduction of a much smaller electron focal spot on the rotating anode, with the first generator of this type (MicroMax-007) being launched by Rigaku at the end of the 1990s. The MicroMax-007 has an effective focal point of only 70 µm diameter at a 6° take-off angle (70 × 700 µm actual size on the anode) and is run at a maximum power of 800 W. For comparison, a typical traditional rotating-anode source has an effective focal point of 300 µm diameter at a similar take-off angle and is run at 3–12 kW. The smaller electron-beam size on the anode not only helps to cool the anode more efficiently but also results in a smaller more dense X-ray beam on the sample. With the narrower beam the number of X-ray photons hitting a small protein crystal is several times higher than was possible before and results in home-laboratory sources with a performance that approaches that of second-generation synchrotron bending-magnet beamlines.

The anode in rotating-anode systems has to be continuously cooled, usually with circulating water, while it spins at high speed (up to 12 000 rev min^−1^). At the same time the inside of the source has to be maintained in high vacuum. These two requirements place a high demand on the quality of the electromechanical components and their assembly. Early rotating-anode generators were prone to failure and required considerable effort to keep them running. More recent systems are more reliable but still require regular maintenance, including the replacement of filaments and ferrofluidic seals and anode polishing.

**Figure 1:**
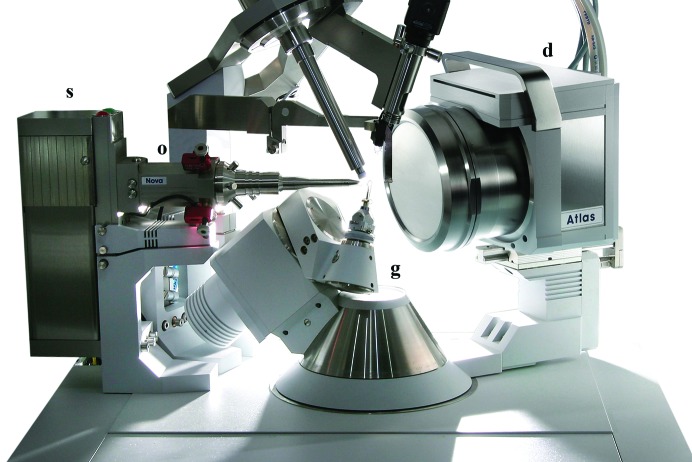
A compact X-ray system consisting of a microfocus sealed-tube source (s), focusing multi-layer optics (o), a four-circle goniometer (g) and a CCD detector (d). Picture courtesy of Agilent Technologies.

In 1990 Uli Arndt suggested that a combination of focusing multilayer mirrors with a small focal spot on the anode in a sealed tube might produce an X-ray beam as powerful as that generated by rotating-anode sources using only a small fraction of the electric power (Arndt, 1990 ▶). He developed a system based on the microfocus sealed tube and demonstrated that such a source, when combined with precise X-ray optics, can indeed produce an intense beam suitable for X-ray data collection from protein crystals (Arndt *et al.*, 1998 ▶). However, several further technological developments were needed before the first commercial systems were successfully launched in 2005/2006. Oxford Diffraction Ltd (now Agilent Technologies) were the first manufacturers of such complete commercially successful X-ray systems based on the new technology. The PX Scanner was designed for analysis of macromolecular crystals *in situ* in crystallization plates and the Xcalibur Nova (later replaced by the SuperNova) was designed for complete data-collection experiments (Fig. 1 ▶). Other manufacturers of X-ray equipment followed suit, including Incoatec (commercialized by Bruker), PANalytical (Rigaku) and Xenocs (MAR Research).

All currently available sealed-tube microfocus X-ray sources share the same basic properties: a small effective electron-beam focus spot on the anode (20–50 µm), very low power requirements (30–50 W), an absence of moving parts and low infrastructure requirements. New sources are still being improved thanks to continued refinement of the multi-layer optics, and systems based on microfocus sealed tubes are becoming a popular choice for many MX laboratories, especially those with good access to synchrotron beamlines. The main benefits of these systems are very low maintenance requirements, low ownership cost and high reliability.

An often overlooked aspect of the home X-ray system is the ability to orient the crystal sample in the X-ray beam. Single-axis rotation cameras do not provide an easy way of precisely orienting a crystal with respect to the beam and rotation-axis directions, which often helps in analyzing crystal symmetry and carrying out more precise measurement of anomalous differences. Most of the current suppliers of sealed-tube microfocus X-ray systems offer three-circle and four-circle goniometers that are fully controlled by the data-collection software and help to achieve more efficient data-collection strategies (Fig. 1 ▶). The value of the ability to orient the crystal in the X-ray beam has recently been recognized by several synchrotron beamlines by the introduction of a variety of ‘mini-kappa’ solutions.

Any development of more powerful X-ray sources for in-house data-collection systems has to address the fundamental issue of the dissipation of the heat generated by the collision of accelerated electrons with the metal target inside the source. For a stationary anode, there is a load limit of about 1 W of power per 1 µm electron-beam diameter on the metal target (Arndt, 1990 ▶). A small focal point means high heat density, and a power density higher than the limit above would damage the anode. To avoid metal melting in high-power rotating-anode generators, the spinning target either has to have a larger radius to increase the relative linear speed of the electron beam in relation to the target surface or it has to rotate faster and be cooled more effectively. Both approaches have been exploited in the design of the latest rotating-anode sources.

**Figure 2:**
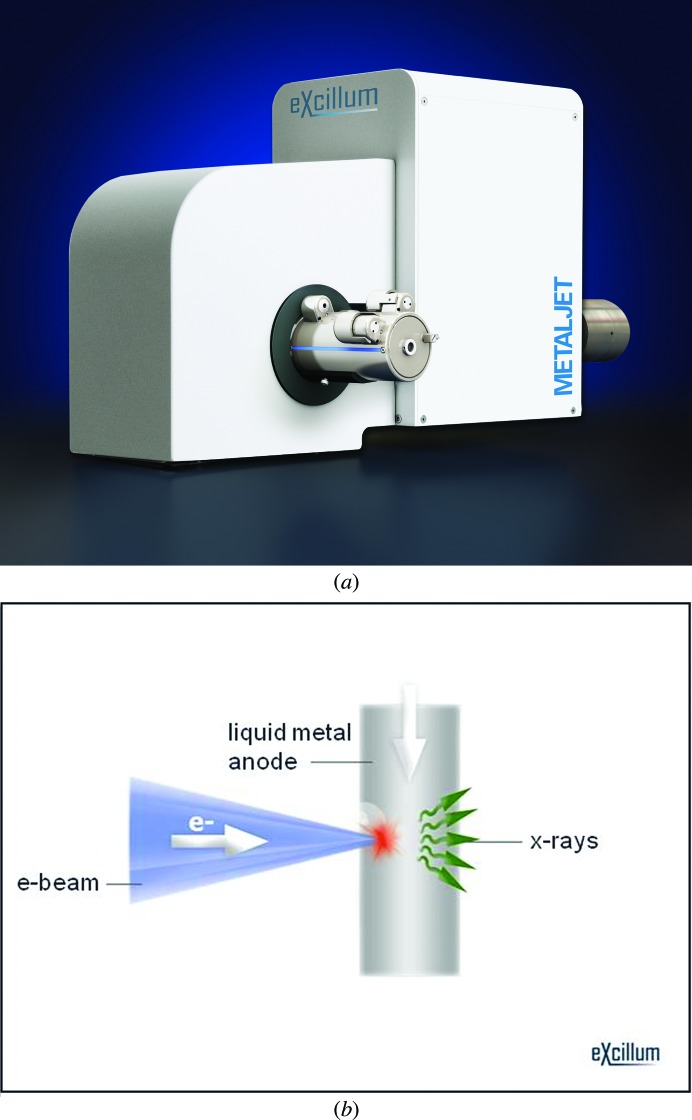
(*a*) Novel X-ray source utilizing a molten gallium anode. (*b*) Schematic principle of the metal-jet technology. Pictures courtesy of Excillum AB.

A novel approach to the melting-anode problem has been developed by Excillum, who replaced the solid metal target with a target that is already molten (!) (Hemberg *et al.*, 2003 ▶). Liquid gallium alloy is pumped in a closed circuit and bombarded by a beam of electrons, producing a very bright X-­ray beam at the Ga *K*α wavelength of 1.34 Å. Fresh liquid target material is supplied at a speed of up to 100 m s^−1^, with the anode load approaching 10 W per 1 µm of electron-beam diameter. The beam flux density in this novel source (Fig. 2 ▶) is expected to exceed the flux density from the most powerful microfocus rotating-anode generators available today.

Developments in X-ray source technology over the last couple of decades, including huge progress in the performance of X-ray optics, has resulted in a tremendous increase in the beam intensity available outside synchrotrons. The performance of a high-end modern microfocus X-ray source equipped with multilayer focusing optics is no worse than that of second-generation bending-magnet synchrotron beamlines not so long ago, and is very similar to that of current rotating-anode generators, with typical exposure times from several seconds to a few minutes per degree. However, precise comparison of the beam intensity for different sources is a difficult and sometimes contentious issue owing to its dependence on a number of factors such as the optics used, the beam profile, the divergence and the measurement aperture. A number of different beam-intensity descriptors are quoted for their products by the manufacturers of X-ray equipment. The nomenclature is often confusing. However, the most commonly used descriptors include beam flux (photons s^−1^), flux density (photons s^−1^ mm^−2^) and brilliance (photons s^−1^ mm^−2^ mrad^−2^). Total beam flux is most often used to describe the properties of a synchrotron beam, while the other measures are usually quoted for home sources. An approximate comparison of the beam brilliance for typical home-laboratory sources is presented in Table 1 ▶, compiled from manufacturers’ websites and own measurements.

## 3. X-ray detectors

Detectors, which are necessary for recording diffraction images during crystallographic data collection, have been developed for both in-house sources and synchrotron beamlines. The larger, more expensive, versions of the main detector types were predominantly made for synchrotrons, while cheaper variants found their way to in-house laboratories. After a long period dominated by the X-ray film, the first electronic detectors become available in the late 1980s and the beginning of the 1990s: multiwire proportional counters (for example, Siemens/Bruker X-1000), television-camera detectors (FAST; developed by Uli Arndt and commercialized by Nonius) and electronically controlled imaging plates (introduced by MAR Research and Rigaku). These devices allowed the automatic transfer of X-ray images to computer storage discs, which greatly sped up the data-collection process and made the handling of the data and their use by data-processing and structure-determination software much easier. Many high-quality data sets were collected using both multiwire and TV detectors; however, each of these technologies had some technical drawbacks that prevented them from gaining a dominant role in MX laboratories. Subsequently, this role was assumed by imaging plates.

Image-plate detectors are made using a plastic sheet containing a photosensitive material that on exposure to X-­rays creates colour centres that can be read out as a digital image in a scanning mode with a laser. They were for many years the most popular choice not only for structural biology home laboratories but also for synchrotron beamlines, owing to their excellent dynamic range, efficiency and large area. The best known examples of imaging plates are the MAR345, produced by MAR Research, and R-AXIS IV^++^, developed by Rigaku. The main drawback associated with imaging plates is the relatively slow readout of the plate by the scanning laser, which ranges from about 1.5 to 4 min depending on the active detector area. While it was not initially seen as a major problem, the long readout became a significant drawback when, owing to the development of brighter microfocus X-ray sources, exposure times shortened significantly. With exposures of several seconds to several minutes per image, a large part of the duty cycle of the image-plate detector was taken up by reading the image, which can markedly slow down the experiment (Muchmore, 1999 ▶).

In the final years of the 20th century and the beginning of the 21st, X-ray detectors based on CCD (charge-coupled device) technology entered the X-ray data-collection arena. Initially, they gained popularity at synchrotron beamlines owing to their drastically shorter readout of diffraction images compared with image plates. This property better suited the short exposure times (seconds rather than minutes) that were becoming the norm at second- and third-generation synchrotron facilities at the time. A CCD detector typically has the shape of a truncated cone, with the wide end covered by a phosphor screen which produces visible light in response to X-­rays. The optical image of the diffraction pattern on the phosphor is then reduced in size by a fibre-optic taper and is projected onto the CCD chip positioned at the narrower end of the detector. Several detector modules (typically four or nine) can be stacked side-by-side in order to provide a larger image area. The multi-chip CCD detectors are heavy, expensive and are used almost exclusively at synchrotron beamlines, although they can be also found at some high-turnover X-ray laboratories in the pharmaceutical industry (for example, at GlaxoSmithKline research laboratories in Stevenage, England and Durham, North Carolina, USA).

For the home source, single-module CCD detectors, which are available with different front-window sizes from about 90 to 165 mm in diameter, are usually preferred (Fig. 1 ▶). Owing to the taper demagnification ratio larger detectors are not as sensitive as smaller ones, so the choice of the appropriate size has to be driven by the working practices of the laboratory. For example, for pre-synchrotron crystal screening a smaller, more sensitive and cheaper version may be quite adequate. Today, a good-quality CCD detector of 135 mm diameter has about four million pixels (2000 × 2000), with pixel size of 0.48 µm, a gain value of 90 e^−^ per photon (for copper radiation) and a readout time from 0.28 s per frame (specification of the Atlas detector from Agilent Technologies). The small pixels in the CCD detectors and the low value of the point-spread function allow efficient data collection at a shorter crystal-to-detector distance than would be possible with image plates and detectors with much larger pixels. Also, the possibility of binning, *i.e.* combining counts from single pixels into larger virtual pixels, allows further control of the detector properties, especially the dynamic range and sensitivity. For example, for a detector with 48 µm CCD pixels a higher dynamic range or a higher sensitivity can be obtained (depending on the binning method) after applying 2 × 2 binning (96 µm virtual pixel area) or 4 × 4 binning (192 µm effective pixel size).

Recently, two other technologies have been used in the design of X-ray detectors: the application of an active-pixel complementary metal-oxide semiconductor (AP CMOS) chip (*e.g.* Bruker Photon100) and hybrid-pixel photon-counting devices (PILATUS series of detectors by Dectris). CMOS technology is not new; it was developed in the 1960s and patented before CCD sensors became available and is the standard technology for constructing integrated circuits in microprocessors, microcontrollers, static RAM and other digital logic circuits. As imaging devices, AP sensors produced by the CMOS process were for many years regarded as inferior owing to their higher noise and lower sensitivity and the need for complicated image corrections. However, they have recently gained enormous popularity as the technology behind many consumer products such as mobile phones, digital cameras *etc*. The main reasons behind this rise in popularity include cheaper manufacturing using simpler production lines, lower power consumption by the chip and the possibility of faster image readout, which made the production of cheaper digital cameras with high-speed high-definition operation possible. However, very sophisticated image-processing software is always used in such devices to correct the non-uniformity and noise issues inherent to AP CMOS technology. It is worth noting that top-end medium-format professional camera manufacturers, such as Mamiya, Pentax and Hasselblad, continue to use CCD imaging chips in their products.

For X-ray detectors, much larger CMOS chips must be used, such as RadEye100, available from Teledyne DALSA, with an active area size of 50 × 100 mm. Since the AP CMOS chip cannot directly record X-rays, a phosphor layer similar to that in CCD detectors is used along with a 1:1 optical coupling. Typically, the chips are used as a pair stacked side by side to create a larger imaging area and are predominantly used in medical radiography (Farrier *et al.*, 2009 ▶). An image-processing circuit combined with a large on-board RAM memory is used to apply the necessary corrections to the diffraction image read from the CMOS chip. The chip is often cooled below 273 K to reduce the readout and dark-current noise. However, cooling to much lower temperature, for example 233 K as used in CCD detectors, is difficult owing to the physical size of the chip and the possibility of its deformation and uneven temperature distribution. In effect, the noise level in CMOS-based detectors is higher than that in CCDs. Since only part of the surface area of each CMOS pixel is used to capture X-ray photons, the overall sensitivity of the CMOS chip is lower. As a result, the detectivity of low X-ray signals is still lower for currently available CMOS detectors than that achievable by the latest CCD-based devices. Excellent diffraction data can be produced by both types of detectors for well diffracting crystals; however, for weak diffractors or short exposure times CCD detectors still have the edge.

Hybrid-pixel photon-counting devices, developed at the Paul Scherrer Institute (Henrich *et al.*, 2009 ▶) and currently used in the PILATUS detectors manufactured by Dectris, offer a different approach similar to the early multiwire detectors. They simply count X-ray photons, without the need to first convert them into visible light. Every X-ray photon is directly converted into an electrical signal by a silicon pixel and counted by the detector system. PILATUS detectors feature a very wide dynamic range (1:1 000 000), a very short readout time (<3.0 ms), no readout noise and a very high counting rate (>2 × 10^6^ counts s^−1^ per pixel), producing a good signal-to-noise ratio. The basic building block of all of the PILATUS detectors is a module with an active area of 83.8 × 33.5 mm and a total of 100 K pixels. The PILATUS 100K is the smallest complete detector system, with only one module, while the PILATUS 6M is a very large device (423 × 434 mm) containing 60 modules. Other sizes are also available. The extremely fast readout makes the larger PILATUS detectors a very attractive proposition for the brightest beamlines, because it allows complete data sets to be collected in a very short time (Trueb *et al.*, 2012 ▶). Also, the shutterless operation of the detector removes errors associated with imprecise synchronization of shutter opening and crystal rotation.

The large-format PILATUS detectors, which are excellent instruments for macromolecular data collection, would be far too costly and impractical to use on in-house X-ray sources. However, the smaller versions of the PILATUS detectors (100K and 300K) available for the home laboratory have some drawbacks resulting from the reduced number of pixels and pixel size (172 × 172 µm), which may impact on the effectiveness of data collection. For example, the 100K version of the detector has only 487 × 195 pixels and may require several detector positions to collect complete data, especially for larger unit cells, owing to limited spatial resolution.

To summarize, a number of area-detector technologies have been developed for single-crystal X-ray diffraction and these vary significantly in both performance and cost. Key factors to consider include the active area, spatial resolution, quantum efficiency, signal detectivity, linearity of response, dynamic range, noise level, speed of operation and cost. Individual characteristics should therefore be considered together, since the detector is a collection of components and features. The best measure of the home-laboratory system performance is the quality of data and the time needed to carry out the data-collection experiment.

## 4. Conclusions

With the huge advances in the technologies applied to macromolecular crystallography in the last decade, the process of diffraction data collection and analysis has undergone a very significant change for the majority of research groups. The wider availability of very high intensity synchrotron beamlines, coupled with much faster data collection and advances in computing infrastructure, have significantly reduced the need for high-end powerful X-ray sources in home laboratories. Many researchers mostly use their in-house facilities to screen crystals and characterize them before synchrotron data collection. Therefore, there is a growing demand for low-maintenance, easy-to-use and effective X-ray systems which can complement synchrotron data collection but can also help to find the optimum crystal-handling procedures, test harvesting and cryoprotecting solutions, select the best crystals for data collection and screen for heavy atoms or ligands. Such systems should also be able to collect high-quality complete data sets for structure determination. In many cases, use of the in-house facility for student teaching is also an important factor.

There is now a variety of X-ray systems on the market that satisfy these needs, including the latest low-power microfocus sources combined with very effective optics and sensitive and efficient detectors. For each research group the choice of the right system will depend on the accessibility of synchrotron beam time (frequency of visits, distance and cost), the type of experiments carried out in the laboratory, the availability of technical support and the funds available, amongst other factors. Relying on just one aspect of the technical specification may lead to an unbalanced system with a lower overall performance. For example, combining a high-flux X-ray source with a less sensitive and slower detector does not give any advantage over a system consisting of a lower powered source equipped with a detector with a several times higher detectivity and a sub-second readout time.

## Figures and Tables

**Table 1:** Approximate X-ray beam brilliance for the main types of in-house sources with optics

System	Power (W)	Actual spot on anode (µm)	Apparent spot on anode (µm)	Brilliance (photons s^−1^ mm^−2^ mrad^−1^)
Standard sealed tube	2000	10000 × 1000	1000 × 1000	0.1 × 10^9^
Standard rotating-anode generator	3000	3000 × 300	300 × 300	0.6 × 10^9^
Microfocus sealed tube	50	150 × 30	30 × 30	2.0 × 10^9^
Microfocus rotating-anode generator	1200	700 × 70	70 × 70	6.0 × 10^9^
State-of-the-art microfocus rotating-anode generator	2500	800 × 80	80 × 80	12 × 10^9^
Excillum JXS-D1-200	200	20 × 20	20 × 20	26 × 10^9^
